# Local health practices and the knowledge of medicinal plants in a Brazilian semi-arid region: environmental benefits to human health

**DOI:** 10.1186/1746-4269-11-11

**Published:** 2015-02-23

**Authors:** Sofia Zank, Nivaldo Peroni, Elcida Lima de Araújo, Natalia Hanazaki

**Affiliations:** Department of Ecology and Zoology, Federal University of Santa Catarina, Campus Universitário Reitor João David Ferreira Lima, Florianópolis, CEP: 88040-900 Santa Catarina, Brazil; Department of Biology, Federal Rural University of Pernambuco, Street Dom Manoel de Medeiros, Recife CEP: 52171-900 Pernambuco, Brazil

**Keywords:** Ethnobotany, Ecosystem health, Human health, Medicinal plants, Local health experts, Etnobotânica, Saúde ecossistêmica, Saúde humana, Plantas medicinais, Especialistas locais de saúde

## Abstract

**Background:**

The concept of eco-cultural health considers the dynamic interaction between humans and ecosystems, emphasizing the implications of the health of the ecosystem for the health and well-being of human populations. Ethnobotanical studies focusing on folk medicine and medicinal plants can contribute to the field of eco-cultural health if they incorporate the perspective and local knowledge of communities. We investigated the local health practices in three rural communities living within the vicinity of a protected area of sustainable use in a semi-arid region of Brazil. We analyzed the opinions of local health experts on the elements that influence human health and on how the environment contributes to this influence. We also analyzed and compared the local knowledge of medicinal plants, as knowledge of this type is an important factor when considering the interaction between environmental and human health.

**Methods:**

We performed structured interviews and free-listings with 66 local health experts. We used content analysis to systematize the elements of the influences on human health. We compared the richness of the plants cited among communities and analyzed the differences among the three communities regarding the ways in which the plants were obtained and the environments in which plants were collected.

**Results:**

The local experts identified several influences of the environment on human health. These influences can be associated with ecosystem services, such as climatic conditions, water and air quality, recreation and medicinal and food resources. We identified 192 medicinal plant species, most of which were gathered from wild ecosystems. The most important environments for the three communities were the plateau mountain and backyards.

**Conclusions:**

The informants had a broad and integrated view of health, perceiving the importance of conserving the environment within the National Forest of Araripe for the health and well-being of the local populations.

## Resumo

**Fundamentação:**

O conceito de saúde eco-cultural considera a interação dinâmica entre os seres humanos e ecossistemas, com ênfase nas implicações da saúde do ecossistema na saúde e bem-estar das populações humanas. Os estudos etnobotânicos sobre medicina popular e plantas medicinais podem contribuir para o campo da saúde eco-cultural, ao inserir a perspectiva e conhecimento local das comunidades. Nós investigamos o processo de saúde em três comunidades rurais que vivem nas proximidades de uma área protegida de uso sustentável em uma região semi-árida do Brasil. Foram analisadas as opiniões dos especialistas locais de saúde sobre elementos que influenciam a saúde humana, e como o ambiente contribui para isso. Foi também analisado e comparado o conhecimento local das plantas medicinais nas três comunidades, um fator importante quando se considera a influência do ambiente na saúde humana.

**Métodos:**

Realizamos entrevistas estruturadas e listagem-livre com 66 especialistas locais de saúde. Utilizamos análise de conteúdo para sistematizar os elementos de influência sobre a saúde humana, comparamos a riqueza das plantas citadas entre as comunidades, e analisamos as diferenças entre as comunidades nas formas de obtenção e nos ambientes de coleta das plantas.

**Resultados:**

Os especialistas locais perceberam diversas influências do ambiente na saúde humana relacionadas aos serviços ecossistêmicos, como condições climáticas, qualidade da água e do ar, recreação, recursos medicinais e alimentícios. Foram identificadas 192 espécies de plantas medicinais, sendo que a maioria é extraída de ecossistemas naturais. Os ambientes mais importantes nas três comunidades foram a serra e os terrenos/quintais.

**Conclusão:**

Concluímos que os informantes possuem uma visão ampla e integrada do processo de saúde, percebendo a importância dos ambientes conservados pela Floresta Nacional do Araripe para a saúde e bem estar das populações humanas.

## Background

Studies of ecosystem health show that the degradation of an ecosystem has adverse effects on human health and well-being; these studies also emphasize the importance of placing human health within an ecological context linked to ecosystem services [1, 2]. Ecosystem services are the benefits that people obtain from ecosystems; they contribute directly and indirectly to the health and well-being of human populations. These benefits can be both material and quantifiable (e.g., food supply, climate regulation, support in nutrient cycling) or intangible, as is the case for cultural services (e.g., recreation, spiritual, aesthetic) [3].

Discussions on the relationship between biological and cultural diversity have also been incorporated into the field of ecosystem health through the concept of eco-cultural health [4]. This concept considers the dynamic interaction between humans and ecosystems, emphasizing the health or pathology of a given ecosystem and the implications of the health of the ecosystem for the livelihoods, health and well-being of human populations.

The relationship between nature and culture becomes more evident in human communities in which there is a greater direct dependence of humans on the environment. In these communities, the health of the ecosystem and the health of the community overlap [5]. This overlap occurs, in part, because the local environment is considered part of the social and cultural identity of the community. Studies related to traditional medicine can provide important insights about the influence of the environment on health and well-being. In folk medicine, health is viewed holistically, including environmental and spiritual elements in the healing process [6, 7]. Medicinal plants, which are obtained from wild environments and through cultivation, are important environmental resources for health-related processes in traditional medicine, especially as home remedies and also for ritualistic purposes. Local health experts are community members who are culture-bearers for folk medicine, and they often maintain important knowledge about environmental and human health [8, 9].

Within this context, ethnobotanical studies focusing on folk medicine and medicinal plants can contribute to the field of eco-cultural health because these studies investigate the process of health care from the perspectives of the communities themselves, with attention to local knowledge, cultural values and practices that enhance the positive relationships between the community and the environment.

In view of the positive effects of a healthy environment on human health, protected areas are potential sites for the development of these studies. As they are legally protected for biodiversity conservation, protected areas can be assumed to be ecologically healthy environments. A healthy environment can be defined as “stable and sustainable, maintaining its organization and autonomy over time” [1].

In this study, we investigated the process of health care in three rural communities living within the vicinity of a protected area of sustainable use in a semi-arid region of Brazil. These communities still depend on natural resources (including plants used for health care) for economic and cultural reasons. Herein, we detail the characteristics of the local health experts who practice folk medicine in these communities. By analyzing the perspectives of local health experts on elements that influence human health, we describe how the environment contributes to the health care context. We also analyze and compared the local knowledge of medicinal plants, which is an important factor when considering the interaction between environmental and human health. Finally, we discuss several implications of protected areas for human health.

### Area of study

**Figure 1:**
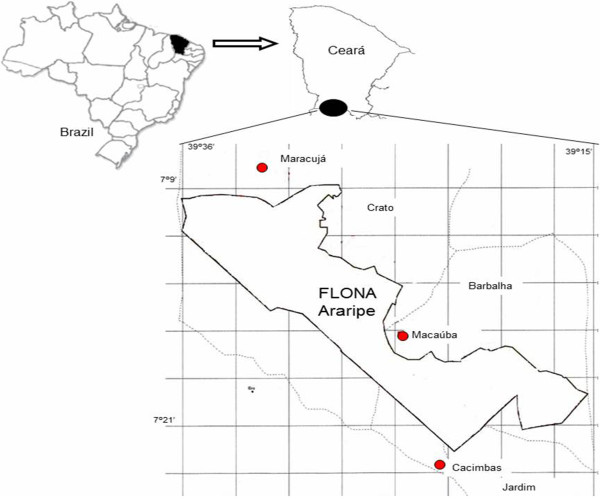
**Rural communities studied in the surroundings of Araripe National Forest (FLONA Araripe), Ceará, Brazil.**

We selected three rural communities in the region of the Araripe within the vicinity of the National Forest (FLONA) of Araripe (Figure 1): Macaúba, Cacimbas and the region of Baixa do Maracujá. This protected area was established in 1946 and was the first national forest created in Brazil. This region is also partially inserted in an Environmental Protection Area (APA of Araripe) that was created in 1997 [10]. The region is within the domain of semi-arid *caatinga* with an island of *cerrado* (Brazilian savanna) vegetation [11]. Araripe is noteworthy for its biological and cultural diversity and has been the subject of ethnobiological studies, e.g., investigations of local pharmacopoeia [12–14] and of the traditional knowledge and management of intensely exploited species [15–17]. We selected three communities that are located close to the National Forest boundaries and that depend on the environment (either for the harvesting of wild species or for the agricultural production of food for family consumption) [12, 13, 17].

Cacimbas and the region of Baixa-do-Maracujá (hereafter called Maracujá) are located in the highlands of a plateau locally known as *serra* (mountains or hills). Both are surrounded by wooded savanna; however, there is also a predominance of *carrasco* (steppe) in Cacimbas, whereas the forested savanna dominates the region of Maracujá. Macaúba is located in the foothills and in the Cariri valley; humid forest (open evergreen forest) and dense *Attalea speciosa* patches are the predominant plant formations in this area.

Cacimbas, also called Horizonte, is 15 km from the center of the city of Jardim and has a population of approximately 260 families [12]. The community is organized into two community-based associations, the Association of Horizonte Residents and the Association of Pequi Collectors; the latter association is most active during the *pequi* (*Caryocar coriaceum*) harvesting season. Approximately 60% of the adults (over 18 years old) of the region are self-described harvesters [12]; the main species extracted in the region are *C. coriaceum* and *Dimorphandra gardneriana*.

Macaúba is located 14 km from the town of Barbalha. This community has a population of approximately 275 families [13] and has two public elementary schools and two Catholic churches that play an important role in the social life of the community. There are two community-based associations in Macaúba: the Association of Women and the Association of Small Farmers. The former association is very prominent in the region; association women from the community meet daily to make oil and coconut jewels from *Attalea speciosa*. Several households depend on this species for their income.

Maracujá comprises three small adjacent settlements: Baixa-do-Maracujá, Cruzeiro and Santo Antônio. We grouped these three sites together because of their geographical proximity and the socio-cultural flow between them. There are approximately 500 families in Maracujá (190 in Cruzeiro, 100 in Baixa-do-Maracujá and 210 in Santo Antônio). These locations are approximately 20 km from the center of the municipality of Crato. There are two public schools, one pre-elementary in Santo Antonio and one elementary school in Cruzeiro. There are four community-based organizations in this region: two residents’ associations in Santo Antonio, and the Resident Association and Women’s Association of Baixa-do-Maracujá. The local residents’ income is derived from the harvesting of plant resources such as *C. coriaceum*, *Hancornia speciosa*, *Himatanthus drasticus* and *D. gardneriana*.

### Environmental and cultural characteristics of the communities

Small rural properties with gardens and backyards near the houses predominated in Macaúba and Maracujá. Cacimbas has an urbanized area in the center of the community, and the houses have small yards (often without crops). Near this center, the houses (small properties with gardens and backyards) are more widely spaced.

All communities have access to the formal health care system (Table 1); in Maracujá, however, the closest health center is 3 km away from the community. Urgent cases are treated in public city hospitals. Access to urban centers is easier in Macaúba, with hourly public buses to downtown Barbalha or Crato. In Maracujá, there are six daily buses to Crato. Cacimbas has no public transportation except for the school bus.

**Table 1:** **Main characteristics of the three studied communities of the Araripe Plateau region, Brazil**

	Macaúba	Cacimbas	Maracujá
Environment	Foothills	Mountain plateau	Mountain plateau
Number of families	275	260	500
Use of the environment	Rural properties	Urbanized center, rural properties	Rural properties, native trees in backyards
Vegetation type	Humid forest and dense *Attalea speciosa* patches	Wooded savanna and *carrasco* (steppe)	Wooded savanna and forested savanna
Distance from the city	15 km	14 km	20 km
Public transport to nearby cities	Hourly buses	Scholar bus	Six buses daily
Access to water	Good: springs and cisterns	Medium: public supply every two days	Scarce: public supply and cisterns
Main religion	Catholic	Catholic	Catholic
Formal health care	One health center Three health professionals	One health center Two health professionals	No health center Four health professionals

Water is a limited resource in the three communities. Macaúba has the most available water, as there are springs in this region of foothills, and several houses have cisterns (Table 1). In Cacimbas, drinking water is publicly supplied every two days. In Maracujá, water availability varies by location; certain households have access to the public water supply, whereas others have cisterns. The main religion in the three communities is Catholicism, but there are syncretisms with African and indigenous religions. Some residents are converting to the Evangelical church.

Due to their location on the plateau, Maracujá and Cacimbas share several similarities, both in relationship to the environment and in relationship to the difficulty of access to urban centers.

## Methods

### Data collection and analysis

#### Identifying local health experts

We interviewed local health experts who act as mediators of the healing process in folk medicine. They were recruited through snowball sampling [18], beginning with suggestions of community leaders and researchers who had previously studied the communities. We conducted a preliminary survey of the names used to designate these experts (e.g., healers, those who offer prayers, midwives, experts on roots, connoisseurs of medicinal plants); these designations were used to seek out new subjects in the snowball sampling process.

We identified four categories of local health experts: prayers/healers (*rezador* or *benzedor*), connoisseurs of medicinal plants, root experts (*raizeiro*) and midwives (*caximbeira* or *parteira*). The prayers or healers are experts who, through blessings, orisons and the use of plants, help in the healing of those who seek them. In the study area, these experts prefer to be called *rezadores* (those who offer prayers). Connoisseurs of medicinal plants are people who are often sought when someone needs a home remedy; they are valued for their knowledge of the plants themselves and their ability to use the plants. The root experts are specialists, located mainly in semi-arid regions, who have specific knowledge of the use of herbal roots. Women who practice or have practiced the craft of midwifery are known locally as *caximbeiras*, while *parteiras* are those who have attended qualifying courses for midwifery in the formal health care system.

### Interviews

We interviewed 66 local health experts (27 in Macaúba, 18 in Cacimbas and 21 in Maracujá). Each local expert was asked a set of previously established questions that included socio-economic indicators and questions on the elements that influence human health. To understand the broad concept of health under the local perspective, we asked two open-ended questions: “What helps us to be healthy? What causes us to be sick?”, and “How do the woods/forests help us to be healthy?”.

We also asked the participants to list the medicinal plants that they knew, including ritual plants (e.g., plants used for protection and blessings), and we asked them to indicate how and where they obtained each species. Plants were collected during walk-in-the-woods tours [19] in backyards, home gardens and areas of native vegetation following standard procedures for ethnobotanical collections [20].

### Data analyses

The collected plants were identified through literature [21, 22], expert consultation with members of the University Cariri Regional (URCA) and the Federal University of Rio Grande do Sul (UFRGS) and by comparison with species in the FLOR herbarium (Federal University of Santa Catarina - UFSC) and in the reference collection of the Laboratory of Human Ecology and Ethnobotany (UFSC). Plants that were not collected were identified by their local names using other studies conducted previously in the region [12, 13]. We followed the APG III classification system, checking the names of the plants against those listed in the database of the Missouri Botanical Garden [23]. The plants collected were registered under the numbers FLOPR0053260 to FLOPR0053263 (FLOR Herbarium, UFSC) and LEHE1727 to LEHE1804 (collection of the Laboratory of Human Ecology and Ethnobotany, UFSC).

Prior informed consent was obtained from all respondents; the project was approved by the Ethics Committee in Research of UFSC and authorized by the Brazilian Environmental Agency (Chico Mendes Institute for Biodiversity - ICMBIO).

We used content analysis [24] to systematize the elements of influence on human health, coding the responses and grouping them according to the themes and patterns identified. The percentages of groups of information were compared among communities. The richness of plants known by each community was analyzed using the method described by Gotelli [25] and the software EstimateS 8.0 [26]. The similarity in medicinal plant knowledge among communities was verified with ANOSIM using the software Primer 6.0 Beta [27]. The differences among the ways of obtaining medicinal plants (wild, cultivated and purchased) and the differences in the environments for plant growth and collection among communities were tested with a chi-square test using PAST software [28]; only the environments cited by all three communities were considered.

## Results and discussion

### Eco cultural aspects of human health

**Figure 2:**
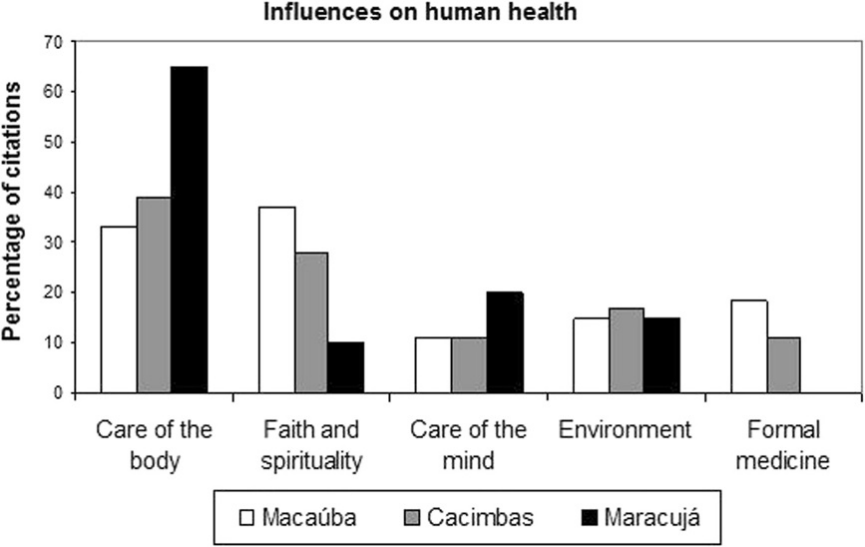
**Percentage of citations for the categories of influence on human health in three communities of the Araripe (Macaúba n = 27, Cacimbas n = 18, and Maracujá n = 21).**

The local experts demonstrated a broad and integrated understanding of human health, realizing that a diversity of factors influences health. From the answers given by the local experts, we identified five groups of influences: 1) Care of the body – 46% of citations (e.g. “Previously the food was healthier, people lived more. Today we are running out for our food with pesticides”, “Today there are people who only work sitting, this harms. Who moves the body has more health”, “Be clean in all that will eat, everything you will do and have everything very clean”); 2) Care of the mind, – 25% of citation (e.g. “Person have peace in life, be calm. Feeling anger and fear takes disease”, “Not caring what people say, do not think too much”); 3) The environment – 15% of citation (e.g. “Do not get out much, stay in the woods. The people who live in woods lives with good health”, “Forest medicine help, I just use forest medicine”); 4) Faith and spirituality – 14% of citation (e.g. “Is to believe in God and Our Lady and pray, who does not pray is dead”, “Pray, asking for healing the Saints, Cicero Priest, and always thank them”) 5) Access to formal medicine – 10% of citation (e.g. “Doctors help, because they have studied hard”, “Having Doctors to serve the community. Here there is just one that comes once a week”) For the question “What helps us to be healthy?”, the influence of elements related to the natural environment were seldom mentioned by the respondents (15% of citation). Elements related to “body care” were the most frequently mentioned (46% of citations), followed by “faith and spirituality” (25%). “Body care” reflects a concern with current issues related to health and food quality, pesticide use, physical exercise and personal hygiene. We found that the distribution of elements perceived to influence health was similar in Macaúba and Cacimbas; in Maracujá, however, “body care” stood out from the other elements (Figure 2). Access to modern medicine was mentioned only in Macaúba and Cacimbas.

The importance of “faith and spirituality” can be explained by the fact that most of the experts interviewed were prayers (70% for Macaúba, 56% for Cacimbas and 52% for Maracujá), for whom faith is critical to the healing process [29, 30]. Macaúba, with the largest number of prayers, had the largest number of citations of elements linked to this group, reflecting the importance of religion. Macaúba is a Catholic community that follows many religious customs, including novenas and Masses transmitted through speakers.

**Figure 3:**
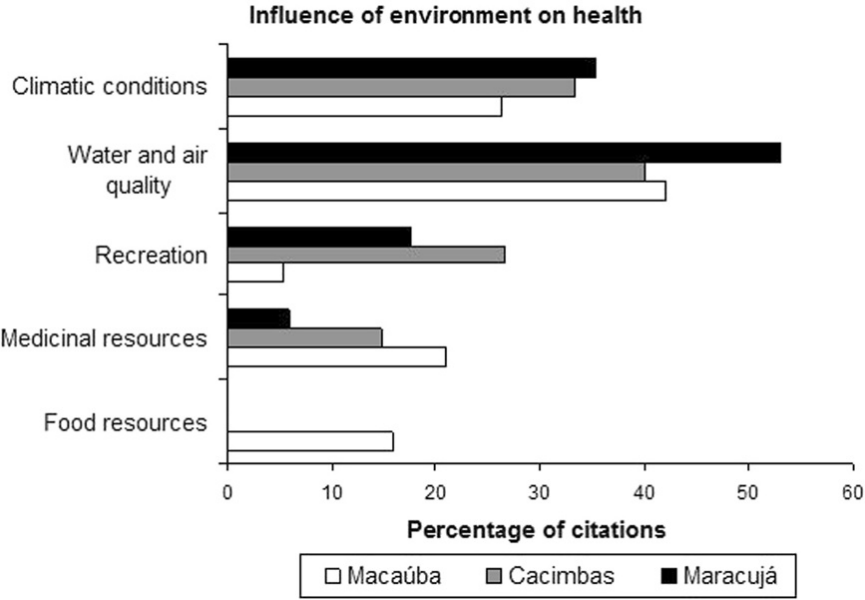
**Percentage of citations for the categories of environmental influence on human health in three communities of the Araripe (Macaúba n = 19, Cacimbas n = 15, and Maracujá n = 17).**

The “environment” category included elements related to the importance of plants used as medicine and welfare-related contact with nature (Figure 2). The influence of the environment on health was detailed through the question “How do the woods/forests help us to be healthy?”. Answers were categorized into five groups: “climatic conditions”, “water and air quality”, “recreation”, “medicinal resources” and “food resources”; the last of these was absent only in Cacimbas (Figure 3). The most common perceptions were related to the “quality of water and air” (average citation of 45%), and comparisons with urban centers were frequently made. For example, one participant noted that “the landscape here has no pollution, the air is good, people feel more healthy” (Female, 40 yrs, Cacimbas). “Climatic conditions” (average citation of 32%) mostly reflected concerns related to rain in this semi-arid region. As one participant noted, “the forest brings rain, without it we do not have winter” (Female, 64 yrs, Maracujá). Forested areas were associated with recreation and medicinal resources; this last characterization was most important in Macaúba (21%). The perceptions of the influence of the environment on human health were linked to ecosystem services of natural areas [3] and demonstrated the importance of regulating services (climate, water purification and air), supply (food and medicinal resources) and cultural services (recreation) in maintaining the health and well-being of the communities studied.

The conservation goals of the National Forest Araripe contribute to the maintenance of climate regulation and water and air cleaning services. Included in the semi-arid area of *caatinga*, the region behaves like an island for vegetation types as *cerrado* (Brazilian savanna) vegetation [11], contributing to a milder climate. The National Forest area is also used for the extraction of medical resources and for cultural ecosystem services, such as recreation. Cultural ecosystem services are intangible and difficult to measure and are often displaced in discussions and research on the benefits of ecosystems [31]. However, understanding the importance of environments for educational and aesthetic enjoyment, recreation, spirituality and other cultural practices is essential for understanding the benefits of environments to human health and the mechanisms that can serve to deepen this analysis in future studies.

The importance of ecosystem services in health and human well-being was highlighted in an article [32], which discusses ecological models of public health that include the natural environment. For the authors is necessary to consider the reciprocal influence of the natural environment on human health and of human actions on ecosystem health. They emphasize the importance to think of ways to apply these concepts of interdependence in health systems. In the region of Araripe, strengthen participatory spaces of decision making, such as the National Forest management council and health councils, to incorporate traditional knowledge can be an efficient way to achieve these goals.

Furthermore, as a category of protected area that enables the sustainable use of a portion of its resources, the establishment of a management structure in partnership with local communities to regulate the extraction of natural resources (including medicinal) and the public use of the National Forest will improve the relationship between environmental health and human health. As discussed by other authors [7], the scientific community needs to consider indigenous peoples and local communities as important allies in the understanding of the complex interrelationship of human health and ecosystem sustainability.

### The local health experts

In the three communities, we recorded a diversity of knowledges and traditional health practices that manifest themselves through local experts. Macaúba had a higher proportion of local health experts per household (0.10) than Cacimbas (0.07) and Maracujá (0.04) (Table 2). Macaúba had also the largest number of prayers (19), while Cacimbas had the only root expert identified in this study. In Maracujá, one of the midwives was considered active and attended courses and midwives’ meetings, while the midwives of the other communities were considered inactive. Several of the experts were found to practice more than one specialty. For example, some prayers were also connoisseurs of medicinal plants or midwives.

**Table 2:** **Characteristics of the local health experts interviewed in Macaúba, Cacimbas and Maracujá**

	Macaúba	Cacimbas	Maracujá
Local experts (Total)	27	18	21
Prayers/healers	19	9	11
Connoisseurs of medicinal plants	8	8	7
Root experts	0	1	0
Midwives	1*	1*	3 (1**)
Local experts/household	27/275 = 0.10	18/260 = 0.07	21/500 = 0.04
Women	78%	72%	86%
Men	22%	28%	14%
Average age (standard deviation)	68 yrs (sd = 13)	62 yrs (sd = 12)	62 yrs (sd = 15)
Occupation			
Farmer	63%	66%	80%
Home care	22%	17%	19%
Harvesting of plants	40%	22%	14%
Retired	48%	33%	38%
Prayers, healers, midwives	7%	11%	10%
Other	7%	11%	10%

*Midwives that are also healers; **Active midwife.

The majority of respondents were women (Table 2), as is the case in other studies on the scope of traditional health, demonstrating that traditional health practices in local communities (including the knowledge of medicinal plants and the prayers) are associated more with women than men [29, 33].

The average age of the respondents was over 60 years (Table 2), confirming that health-related knowledge is generally the province of the older members of the population [29, 34–36]. This characterization reinforces the importance of maintaining and enhancing the ways and means of transmitting traditional knowledge and allowing the acquisition of knowledge by new generations.

The main occupation of the respondents from the three communities was agriculture (Table 2). Macaúba had the highest percentage of respondents who harvested wild plants (40%), followed by Cacimbas (22%) and Maracujá (14%). These activities demonstrate the connection between most respondents and the environment, whether through the practice of agriculture or the extraction of wild species. Approximately 10% of the respondents cited traditional health practices as an occupation (Table 2).

### Medicinal plants and environments used for plant gathering

**Figure 4:**
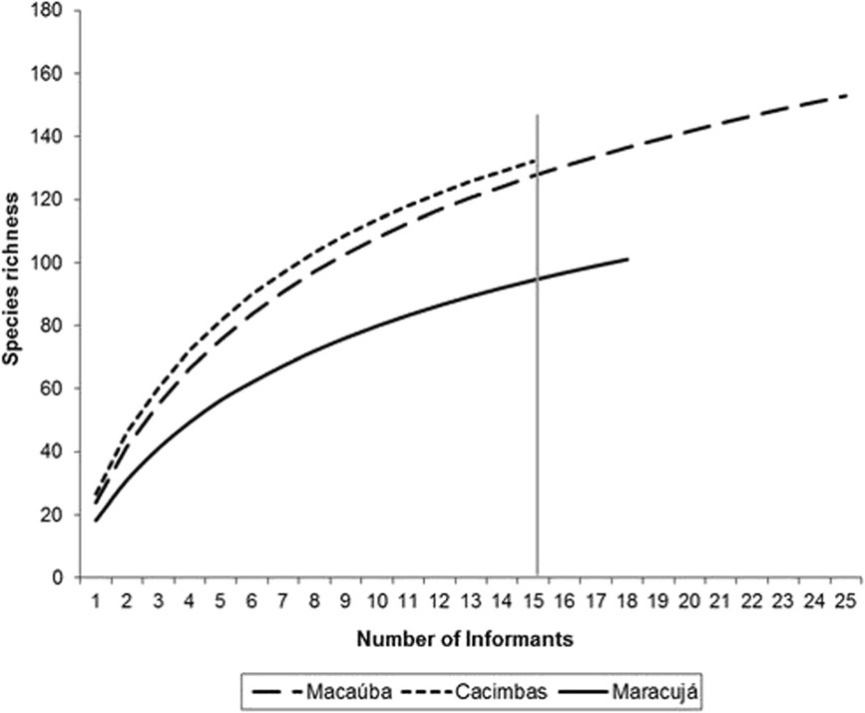
**Expected richness (rarefaction curve) comparing the richness of medicinal plants in three communities of the Araripe (Macaúba n = 25, Cacimbas n = 15, Maracujá n = 18).** Confidence intervals (CI) at 95% for n = 15: Macaúba 139 > CI_95%_ > 116; Cacimbas 142 > CI_95%_ > 122; and Maracujá 104 > CI_95%_ > 85.

The cultivation and the harvesting of wild plants include an array of medicinal species associated with health practices. We identified 192 species of medicinal plants, both native and cultivated. Macaúba had the highest species richness (153 species), followed by Cacimbas (132 species) and Maracujá (101 species). The expected richness of species of Macaúbas and Cacimbas did not differ, while Maracujá had a significantly lower expected richness (Figure 4). The diversity and richness of medicinal plants are important indicators when considering eco-cultural health [4], and are essential for the robustness of the eco-cultural system, which rely on these resources. The medicinal plants collaborates directly to the people’s health when used for the treatment of different diseases, and indirectly to contributes to the resilience of eco-cultural systems [4].

Among the 20 most cited plants (cited by more than 30% of the respondents in at least two communities) 10 were cultivated species and 10 were harvested/extracted (Table 3); five wild species and six cultivated species were mentioned in more than 40% of the citations. These species include *Scoparia dulcis*, *M. urundeuva*, *Himatanthus drasticus*, *Hymenea stigonocarpa*, *Stryphnodendron coriaceum*, *Mentha* sp 1, *Plectranthus amboinicus*, *Rosmarinus officinalis*, *Ruta graveolens*, *Lippia alba* and *Kalanchoe pinnata. R. graveolens* and *S. dulcis* are used for both medicinal and ritualistic purposes for healing and protection by prayers. *H. drasticus*, *M. urundeuva*, *H. stigonocarpa* and *S. coriaceum* are wild species extracted for both family use and for sale at fairs in the region. Of these, *H. drasticus* is the only species that has regulated harvesting in the area of the National Forest. *M. urundeuva* is one of the most popular medicinal plants of *Caatinga*[37] and is commonly found in the pharmacopoeia of rural communities of semiarid environments. *Mentha* sp. 1, *P. amboinicus*, *R. officinalis*, *L. alba*, *R. graveolens* and *K. pinnata* are cultivated exotic plants that are also common elsewhere in the semiarid regions [37, 38]. A prevalence of common exotic species in the local pharmacopoeias has also been observed in other regions in Brazil and Latin American countries [39]. However, the knowledge and use of these plants does not reduce the importance of the native/extracted plants; instead, these introduced plants expand the range of possible treatments [40, 41].

**Figure 5:**
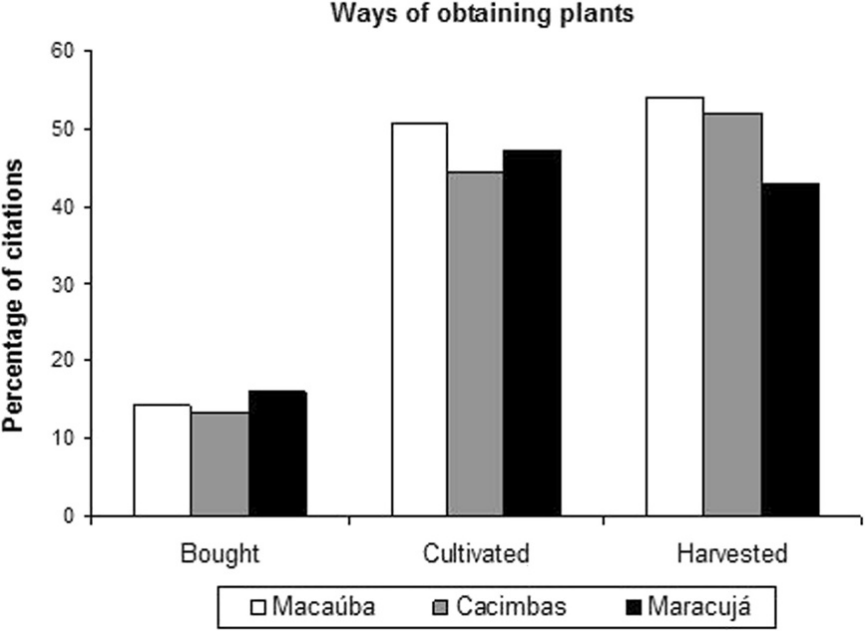
**Ways of obtaining medicinal plants (bought, cultivated and harvested from the wild) in the three communities studied in the Araripe (Macaúba n = 153, Cacimbas n = 132 and Maracujá n = 101).**

The connections between the healers and the plants depend on several environments surrounding the communities. Local health experts obtain medicinal plants through cultivation in their backyards, harvesting from natural ecosystems and purchasing at fairs and markets in the region (Figure 5). In Macaúba and Cacimbas, harvesting is the main form of production (54% and 52%, respectively); in contrast, cultivation predominates in Maracujá (47%). The method of obtaining plants differed significantly between Maracujá and Cacimbas (p <0.001) and between Maracujá and Macaúba (p <0.001) but not between Macaúba and Cacimbas.

**Table 3:** **Percentage of citations of the main medicinal plant species (for plants cited by at least 30% of two communities)**

	Botanical name	Family	Env	MA	CA	BA	Average
Cultivated species	*Mentha* sp1.	Lamiaceae	B	83	60	89	77
	*Plectranthus amboinicus* (Lour.) Spreng.	Lamiaceae	B	83	60	89	77
	*Ruta graveolens* L.	Rutaceae	B	46	80	61	62
	*Rosmarinus officinalis* L.	Lamiaceae	B	50	60	61	57
	*Kalanchoe pinnata* (Lam.) Pers.	Crassulaceae	B	42	53	50	48
	*Lippia alba* (Mill.) N.E.Br. ex Britton & P.Wilson	Verbenaceae	B	58	33	44	45
	*Citrus sinensis* (L.) Osbeck	Rutaceae	B	58	33	17	36
	*Aloe vera* (L.) Burm.f.	Xanthorrhoeaeceae	B	42	33	17	31
	*Ocimum gratissimum* L.	Lamiaceae	B	33	33	6	24
	*Alpinia zerumbet* (Pers.) B.L. Burtt & R.M. Sm.	Zingiberaceae	B	38	0	27	22
Harvested wild species	*Stryphnodendron coriaceum* Benth.	Fabaceae	C; F; P; B	38	60	50	49
	*Himatanthus drasticus* (Mart.) Plumel	Apocynaceae	C; F P; B	42	60	44	49
	*Scoparia dulcis* L.	Plantaginaceae	SF; B	63	53	22	46
	*Hymenaea stigonocarpa* Mart. ex Hayne	Fabaceae	C; Caa; P; F;B	54	53	22	43
	*Myracrodruon urundeuva* Allemão	Anacardiaceae	C; F; P	50	40	33	41
	*Chenopodium ambrosioides* L*.*	Chenopodiaceae	B	48	27	50	40
	*Jatropha gossypiifolia L.*	Euphorbiaceae	SF; B	46	60	11	39
	*Hancornia speciosa* Gomes	Apocynaceae	C; P; B	33	40	39	37
	*Hybanthus calceolaria* (L.) Oken	Violaceae	F; S; B	17	33	44	32
	*Coutarea hexandra* (Jacq.) K. Schum.	Rubiaceae	C; Caa; F; P	38	33	6	26

Env = environment of occurrence; MA = Macaúba; CA = Cacimbas; BA = Maracujá; P = mountain plateau; F = foothills; C = *Cariri*; B = Backyards; SF = Secondary forest; Caa = *Caatinga.*

Backyards are important places for obtaining these most-cited medicinal plants, and eight of 10 wild species also occur in these managed areas (Table 3), showing that medicinal plants are maintained and favored in areas close to the dwellings of these managed environments. Macaúba and Cacimbas had more species in common than Maracujá. This result was significant for both cultivated and wild species (Table 4). Cacimbas and Macaúba shared 43 species that were not cited in Maracujá. Ten species had an average percentage of citation of more than 20% (Table 5). Most of these species are wild, including *Centrosema* sp., *Croton* sp 1, *Amburana cearensis*, *Erythroxylum vaccinifolium*, *Anadenanthera colubrina*, *Astronium fraxinifolium*, *Aristolochia* sp. and *Dorstenia brasiliensis*. Some of these plants are commonly encountered in the *Caatinga*, including *A. colubrina*, *A. cearensis* and *Croton* sp 1; the first two of these are very popular in folk medicine in northeastern Brazil [37]. *Centrosema* sp., *E. ampliofolium*, *A. fraxinifolium*, *Aristolochia* sp. and *D. brasiliensis* are species that, according to the informants, grow in the plateau and foothills of Araripe. The communities were similar in some aspects of medicinal plants and also showed specificities, both of which should be considered in future actions related to human health and the environment.

**Table 4:** **Similarity between cited plants in each community (ANOSIM)**

Wild/Cultivated	Macaúba	Cacimbas	Maracujá
Macaúba		0.4 (W)	**0.01** (W)
Cacimbas	0.08 (C)		**0.02** (W)
Maracujá	**0.002** (C)	**0.001** (C)	

Bold values reflect significant differences for p < 0.05. W = wild; C = Cultivated.

**Table 5:** **Percentage of citations of exclusive species in Macaúba and Cacimbas**

Species	Macaúba	Cacimbas	Average
***Centrosema*** **sp.**	28	33	31
***Croton*** **sp1.**	28	33	31
***Amburana cearensis*** **(Allemão) A.C. Sm.**	20	33	27
*Heliotropium indicum* L.	28	20	24
***Erythroxylum vaccinifolium*** **Mart.**	8	40	24
*Allium sativum* L.	20	27	23
***Anadenanthera colubrina*** **(Vell.) Brenan**	32	13	23
***Astronium fraxinifolium*** **Schott ex Spreng.**	32	13	23
***Aristolochia*** **sp.**	16	27	21
***Dorstenia brasiliensis*** **Lam.**	28	13	21

Wild species are in bold.

The predominance in the knowledge and use of native species in arid and semi-arid regions can be explained by the seasonality hypothesis [40], according to which there is a preference for native resources that are always available. The use of medicinal plants grown in arid environments is limited by irregular rainfall and the need to save water for other more important uses [40]. Therefore, these human communities depend more on medicinal resources of native ecosystems, making it clear the importance of the health of these ecosystems for maintaining the health of surrounding communities.

The five environments where harvested plants are grown are characterized by the relief features of the region: 1) the *Cariri* valley, 2) foot hills or slopes of the mountain, 3) plateau mountain or forest, 4) *Caatinga* or savanna hinterland and 5) terrains or backyards. The *Cariri* valley refers to the northeastern portion of the Araripe region, where rainfall is more pronounced than in the southwestern region of savanna hinterlands [14].

**Figure 6:**
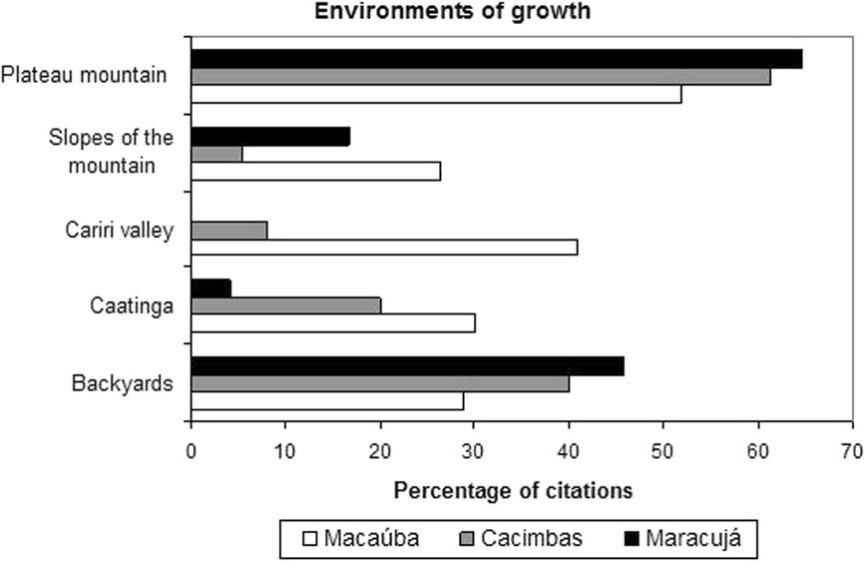
**Places in which medicinal plants extracted from or grown in the three communities studied in the Araripe were found (Macaúba n = 153, Cacimbas n = 132 and Maracujá n = 101).**

The mountain plateau is the environment where most of the medicinal plants cited by the three communities grow (Figure 6); this finding was expected in Maracujá and Cacimbas, as these two communities are located on the mountain plateau. In Macaúba, 22% of local health experts had lived in the region of the mountain plateau, which may explain their knowledge of the flora of this region. Most of the vegetation of the mountain plateau is protected by the National Forest, and several species grow mainly in the protected area, which demonstrates the importance of maintaining this area as a reservoir of medicinal plants. However, there are still no mechanisms in place to regulate the extraction of these plants (except for *H. drasticus*); as a result, many species end up being extracted from the protected area without the authorization of the environmental agency.

Macaúba had higher percentages of *Cariri* and foothills than the other two communities (Figure 6), most likely due to its closer location to these environments. The sites of growth and collection differed significantly among the three communities (p <0.001).

The *Caatinga* (or hinterland) was cited more than 20% of the time in Macaúba and Cacimbas, reflecting the importance of this environment for the pharmacopeia of these communities. In addition, 17% of respondents in Cacimbas, 11% in Macaúba and 5% in Maracujá had lived in the area of *Caatinga* hinterlands. Plants from these environments are easily obtained at local markets and are commonly encountered in the nearby cities, which shows the importance of these spaces in maintaining the knowledge and use of herbal therapies [37, 42].

From the results, we realize the importance of different environments to obtain medicinal plants, and only the mountain plateau is protected by National Forest. Thus, is important to design strategies for conservation of environments not protected by the National Forest, considering that the capacity to maintain the diversity found in different landscapes is also and important element for eco-cultural health [4].

### Reflections on ecosystem health and human health in the Araripe region

The local health systems of Araripe, as mediated by the local health experts, maintain a positive linkage between the community and the environment. This linkage can be observed for both the ecosystem benefits that are perceived by the local health experts and their knowledge and use of herbal therapies.

The existence of the National Forest Araripe (FLONA) contributes to the health of the surrounding communities, both at the level of prevention, through the provision of ecosystem services, and for obtaining medicinal plants used in the preparation of home remedies. The establishment of guidelines may be necessary to formalize the extraction of plants for domestic use within the protected area [16, 17], but it is important to consider that the scale of extraction for domestic use is quantitatively smaller than the extraction for commercial and industrial uses.

From an eco-cultural health perspective, it is essential to consider the governance of the commons [43]. Strengthening the participatory management of these protected areas can improve the conservation of nature with the promotion of the health and well-being of the surrounding communities, especially because the local view of health incorporates both plant resources and different environments of the semi-arid region. Furthermore, it is important that the scientific community consider local knowledge to develop a more holistic and transdisciplinary research approach directed toward both ecosystem sustainability and human health.

## Conclusions

The communities studied have access to the formal health system but also maintain local knowledge and health practices that establish a positive relationship with the environment. The local health experts have a broad and integral view of different elements that influence human health, such as spirituality, care of the body and mind, the environment and the formal health system. We noticed that the main keepers of knowledge and practices related to traditional health practices were elder women, revealing the importance of initiatives that collaborate to the empowerment of these women, enhancing and disseminating this knowledge and allowing its continuity.

We found that a rich variety of medicinal plants, both wild species and cultivated species, are known in the studied communities. These plants are central to the local practices related to health care. We noted that the mountain plateau environment and backyards were the most important places for collecting medicinal plants, reinforcing the contribution of the environment to the health care context. The presence of the protected areas contributes to the health and well-being of the surrounding communities in this semi-arid region of Brazil, both through the ecosystem services provided by these protected areas and through their role as a reservoir for species used for medicinal purposes. We suggest that further studies can add in-depth analyses about the relationship between local practices, ethnobotanical knowledge and eco-cultural health and also about the role of protected areas in the health of local communities.

## Abbreviations

FLONA: National forest
APA: Environmental protection area
UFSC: Federal University of Santa Catarina
URCA: University Cariri Regional
UFRGS: Federal University of Rio Grande do Sul
ICMBIO: Chico Mendes Institute for Biodiversity.
